# ROS Regulate Cardiac Function via a Distinct Paracrine Mechanism

**DOI:** 10.1016/j.celrep.2014.02.029

**Published:** 2014-03-20

**Authors:** Hui-Ying Lim, Weidong Wang, Jianming Chen, Karen Ocorr, Rolf Bodmer

**Affiliations:** 1 Development, Aging and Regeneration Program, Sanford-Burnham Medical Research Institute, La Jolla, CA 92037, USA; 2 Free Radical Biology and Aging Program, Oklahoma Medical Research Foundation, Oklahoma City, OK 73104, USA; 3 Immunobiology and Cancer Program, Oklahoma Medical Research Foundation, Oklahoma City, OK 73104, USA; 4 Key Laboratory of Marine Biogenetic Resources, The Third Institute of Oceanography, State Oceanic Administration, Xiamen, Fujian 361005, China

## Abstract

Reactive oxygen species (ROS) can act cell autonomously and in a paracrine manner by diffusing into nearby cells. Here, we reveal a ROS-mediated paracrine signaling mechanism that does not require entry of ROS into target cells. We found that under physiological conditions, nonmyocytic pericardial cells (PCs) of the Drosophila heart contain elevated levels of ROS compared to the neighboring cardiomyocytes (CMs). We show that ROS in PCs act in a paracrine manner to regulate normal cardiac function, not by diffusing into the CMs to exert their function, but by eliciting a downstream D-MKK3-D-p38 MAPK signaling cascade in PCs that acts on the CMs to regulate their function. We find that ROS-D-p38 signaling in PCs during development is also important for establishing normal adult cardiac function. Our results provide evidence for a previously unrecognized role of ROS in mediating PC/CM interactions that significantly modulates heart function.

## INTRODUCTION

Reactive oxygen species (ROS), including hydrogen peroxide (H_2_O_2_) and superoxide anions (O_2_^−^), are highly reactive molecules produced by the incomplete reduction of oxygen, and their production is typically associated with disease pathogenesis. However, it is now recognized that moderate amounts of ROS can act as signaling molecules to modulate normal cellular processes (Covarrubias et al., 2008; Dröge, 2002). Studies of the physiological and pathophysiological effects of ROS signaling have classically focused on cell-autonomous signaling, in which intracellular production of ROS induces changes in the ROS-generating cell (Owusu-Ansah and Banerjee, 2009; Thannickal and Fanburg, 2000). More recently, evidence suggests that ROS could serve as paracrine signaling mediators upon pathological stimulation (Love et al., 2013; Niethammer et al., 2009; Wu et al., 2012). For instance, in response to tissue damage, wound-derived H_2_O_2_ diffuses into nearby neutrophils and acts in these cells to direct their recruitment to the wound (Niethammer et al., 2009; Yoo et al., 2011). A paracrine role of ROS-mediated signaling in the control of tissue physiology is currently unclear and is the central theme of investigation in this study.

Paracrine communication between neighboring cells and the surrounding extracellular matrix (ECM) enables cells within a tissue to position and coordinate their functions, features that are critical for maintaining tissue homeostasis. In the human heart, which comprises a broad array of cell types, signaling pathways within myocytes and crosstalk between myocytes and nonmyocytes play crucial and interdependent roles in ensuring that the heart responds appropriately to physiological and pathological stimuli (Tian and Morrisey, 2012; Tirziu et al., 2010). For example, paracrine signaling from the epicardium and endocardium through pathways such as fibroblast growth factor- and retinoic acid-dependent signaling is critical for proper growth and differentiation of the myocardium (Brade et al., 2011; Merki et al., 2005). Although paracrine interactions between myocytes and nonmyocytes play important roles in the proper development and function of the myocardium, the underlying mechanisms remain poorly understood.

**Figure 1:**
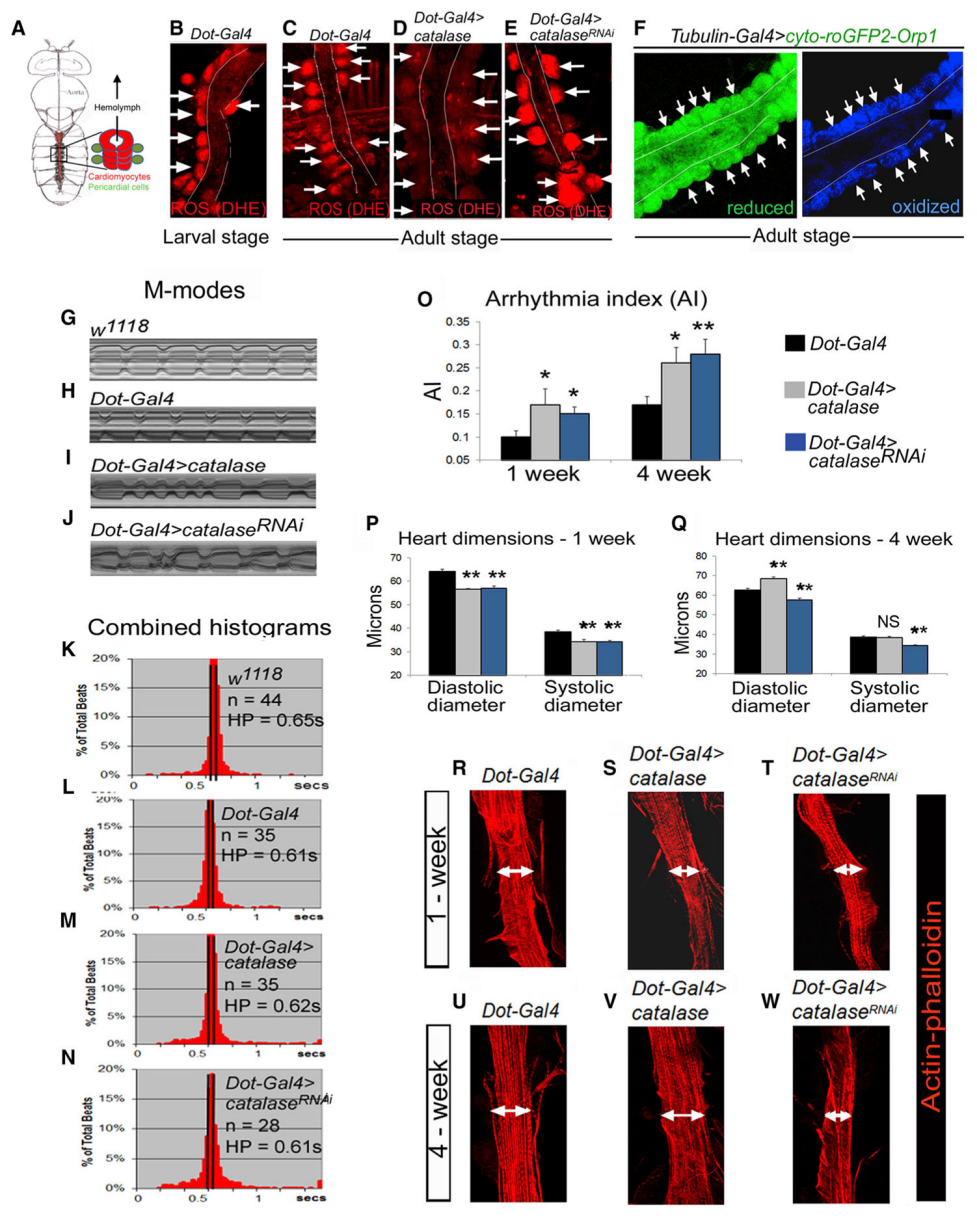
The PCs of the *Drosophila* Heart Contain Increased Levels of ROS Compared to Adjacent CMs that Affect Heart Structure and Function (A) Schematic of an adult *Drosophila* heart depicting two central rows of CMs (red) surrounded by parallel rows of PCs (green). (B–E) DHE fluorescence in control third-instar larval hearts (B), control adult hearts (C), and hearts from adult flies with PC-specific (*Dot-GAL4* driver) *catalase* overexpression (D) or *catalase* RNAi (*catalase^RNAi^*) (E). Arrows indicate increased and decreased fluorescence in PCs compared with the adjacent CMs. (F) Whole-heart expression (*tubulin-GAL4* driver) of cyto-roGFP2-Orp1 showing ubiquitous expression of the reduced form (left; green) and detectable levels of the oxidized form only in PCs (right; blue). (G–J) Representative 5 s M-mode traces showing movement of heart tube walls (y axis) versus time (x axis) for hearts from 1-week-old flies with the indicated genotypes. w*^1118^*, wild-type. (K–N) Combined histograms showing the distribution of HPs for 1-week-old flies. n, number of flies. (O–Q) AI (O) and heart dimensions (P and Q) in 1- and 4-week-old flies. All error bars indicate SEM. *p < 0.05 and **p < 0.01 compared with *Dot-GAL4* controls by two-tailed paired t test and one-way ANOVA. NS, not significant. (R–W) Representative confocal images of phalloidin staining (filamentous actin; red) of fixed heart preparations from 1-week-old (R–T) and 4-week-old (U–W) flies of the indicated genotypes. Double-headed arrows indicate similar regions of the heart. Anterior is to the top. See also Figures S1–S4.

The *Drosophila* heart is a linear tube made up of two central rows of cardiomyocytes (CMs) surrounded by nonmyocytic pericardial cells (PCs) (Figure 1A). *Drosophila* PCs are known to critically influence myocardial development and postnatal heart function (Buechling et al., 2009; Fujioka et al., 2005), similar to the crucial role played by intercellular signaling between myocytes and nonmyocytes in the mammalian heart. Using a combination of genetic and imaging approaches, we found higher concentrations of ROS in PCs than in CMs under physiological conditions. The genetic alteration of ROS levels to sub- or supra-physiological levels in PCs adversely affects cardiac rhythm and morphology, suggesting that ROS in PCs act in a paracrine manner to regulate normal cardiac function. We showed that genetic down- or upregulation of ROS levels in the PCs does not alter the levels of ROS in CMs. Moreover, similar manipulations of ROS-metabolizing enzymes in the CMs do not have any effect on cardiac function. Taken together, these results indicate that ROS do not diffuse from PCs into CMs to exert their function, but rather, ROS control the production of downstream signals in PCs that act in a paracrine manner on CMs to regulate their proper function. Furthermore. we identified that ROS activate downstream D-MKK3-D-p38 signaling in PCs that in turn directs normal cardiac function and that ROS-D-p38 signaling in PCs during development plays an important role in establishing normal adult cardiac function.

## RESULTS

### Elevated Levels of Physiological ROS in PCs versus in CMs

To determine whether ROS could participate in the crosstalk between PCs and CMs that has been recognized to be important for heart physiology in *Drosophila* (Buechling et al., 2009; Fujioka et al., 2005), we first examined the levels of endogenous ROS in the heart under physiological conditions. We probed live *Drosophila* heart tissues with the membrane-permeable dye, dihydroethidium (DHE), in which oxidation by endogenous ROS, particularly O_2_^−^, generates stable fluorescent products (Owusu-Ansah and Banerjee, 2009). We found that DHE fluorescence was stronger in PCs than in the adjacent CMs (Figures 1B and 1C), indicating the presence of higher levels of endogenous ROS in PCs than in CMs. Furthermore, we used a genetically encoded fluorescent redox reporter, cyto-roGFP2-Orp1 (Albrecht et al., 2011), in conjunction with the bipartite *GAL4/UAS* system (Brand and Perrimon, 1993), to assess the endogenous state in PCs and CMs. Orp1-mediated oxidation, particularly by H_2_O_2_, induces a conformational change in the linked roGFP2 variant that changes its fluorescence from 488 to 405 nm excitation (Albrecht et al., 2011). Consistent with the results of the DHE experiments, we found higher levels of oxidized roGFP2-Orp1 in PCs than in CMs (Figure 1F). In addition to ROS detection, we also examined cardiac expression of a GFP construct driven by the promoter of the ROS response gene *glutathione S-transferase D1* (*GSTD1*) (Sawicki et al., 2003). We found a higher expression of *GSTD1-GFP* (Sykiotis and Bohmann, 2008) in PCs than in CMs (Figures S1A and S1B). Collectively, these observations demonstrate that under physiological conditions, ROS levels are higher in PCs than in CMs and, therefore, might serve as paracrine signals to regulate heart function.

### Pericardial ROS Are Essential for Maintaining Normal Cardiac Function in a Paracrine Manner

To investigate the role of pericardial ROS on cardiac function, we decreased ROS levels specifically in PCs by using a PC-specific *Dot-GAL4* driver to overexpress the H_2_O_2_-degrading enzyme, catalase (Zámocký et al., 2012). In flies that overexpress catalase specifically in their PCs (*Dot-GAL4>catalase*), the levels of physiological ROS were significantly reduced (Figures 1D, S1C, S1E, and S1F). To assess the functionality of adult hearts with pericardial overexpression of catalase, we used the Semi-automatic Optical Heartbeat Analysis (SOHA) method to precisely quantify heart contractility parameters in semi-intact adult fly heart preparations (Ocorr et al., 2009). Compared to wild-type (*w^1118^*) and control (*Dot-GAL4*) hearts, the *Dot-GAL4>catalase* hearts exhibited a marked deterioration of the regularity of heart rhythm, as shown by the increased frequency of irregular beats (Figures 1G–1I), broader distribution of heart periods (HPs; heartbeat length) (Figures 1K–1M), and significantly increased arrhythmia index (AI; SD of the HP, reflecting beat-to-beat variation; Fink et al., 2009) (Figure 1O). Further indicating cardiac dysfunction, flies with PC-expressed catalase exhibited a narrowed heart tube phenotype at 1 week of age, as manifested by significant decreases in the diastolic and systolic diameters compared with control hearts (Figures 1P, 1R, and 1S). Interestingly, by 4 weeks of age, the flies with reduced pericardial ROS levels had enlarged heart tubes compared with control hearts because of significantly greater diastolic diameters (Figures 1Q, 1U, and 1V). Thus, lowering ROS levels in PCs induced dynamic remodeling of the myocardial tube, consisting of an initial thinning followed by progressive dilation. These cardiac deficits were also observed when catalase was overexpressed with another PC-specific *GAL4* driver, *Sns-GCN-GAL4* (Zhuang et al., 2009) (Figures S2A–S2C, S2F, S2G, S2J, and S2K). In a second approach to reduce pericardial ROS levels, we overexpressed another antioxidant enzyme, superoxide dismutase (SOD), which catalyzes O_2_^−^ detoxification (Miller, 2012). Similar to the effects of catalase, PC-specific overexpression of both the cytosolic and mitochondrial isoforms of SOD (SOD1 and SOD2) increased the incidence of cardiac arrhythmia and caused constriction of the heart tube (Figures S3A, S3B, S3D, S3E, and S3G–S3I). Thus, reducing ROS levels specifically in PCs adversely affects myocardial function and morphology.

We next asked whether increasing ROS levels in PCs would also affect cardiac function. First, ROS levels were increased systemically by feeding flies with the oxidative stress-generating agents, paraquat and H_2_O_2_. Both agents elicited a significant increase in the AI and a tendency toward thinner heart tube dimensions (Figures S4A and S4B). Next, ROS levels were specifically increased in PCs by RNAi-mediated knockdown (KD) of *catalase* (Figures 1E, S1D, S1E, and S1G). Interestingly, PC-specific elevation of ROS levels also caused an abnormal heart rhythm, similar to that observed following PC-specific reduction of ROS levels; namely, increased frequency of irregular beats, broader distribution of HPs, and significantly increased the AI (Figures 1J, 1N, 1O, S2D, and S2H) compared with wild-type and control hearts (Figures 1G, 1H, 1K, 1L, 1O, S2B, and S2F). Of note, PC-targeted KD of *catalase* caused cardiac narrowing with significant reductions in both the diastolic and systolic diameters of young and old flies compared with control flies (Figures 1P–1R, 1T, 1U, 1W, and S2K). Moreover, PC-specific KD of both *SOD1* and *SOD2* also caused cardiac phenotypes similar to those of the pericardial *catalase* KD flies (Figures S3C and S3F–S3I). Taken together, these data demonstrate that supra- or subphysiological levels of ROS in PCs adversely affect cardiac rhythm and morphology, suggesting that physiological levels of ROS within PCs are critical for their paracrine role in maintaining normal heart function.

### No Evidence for Diffusion of ROS from PCs into CMs to Control Cardiac Function

**Figure 2:**
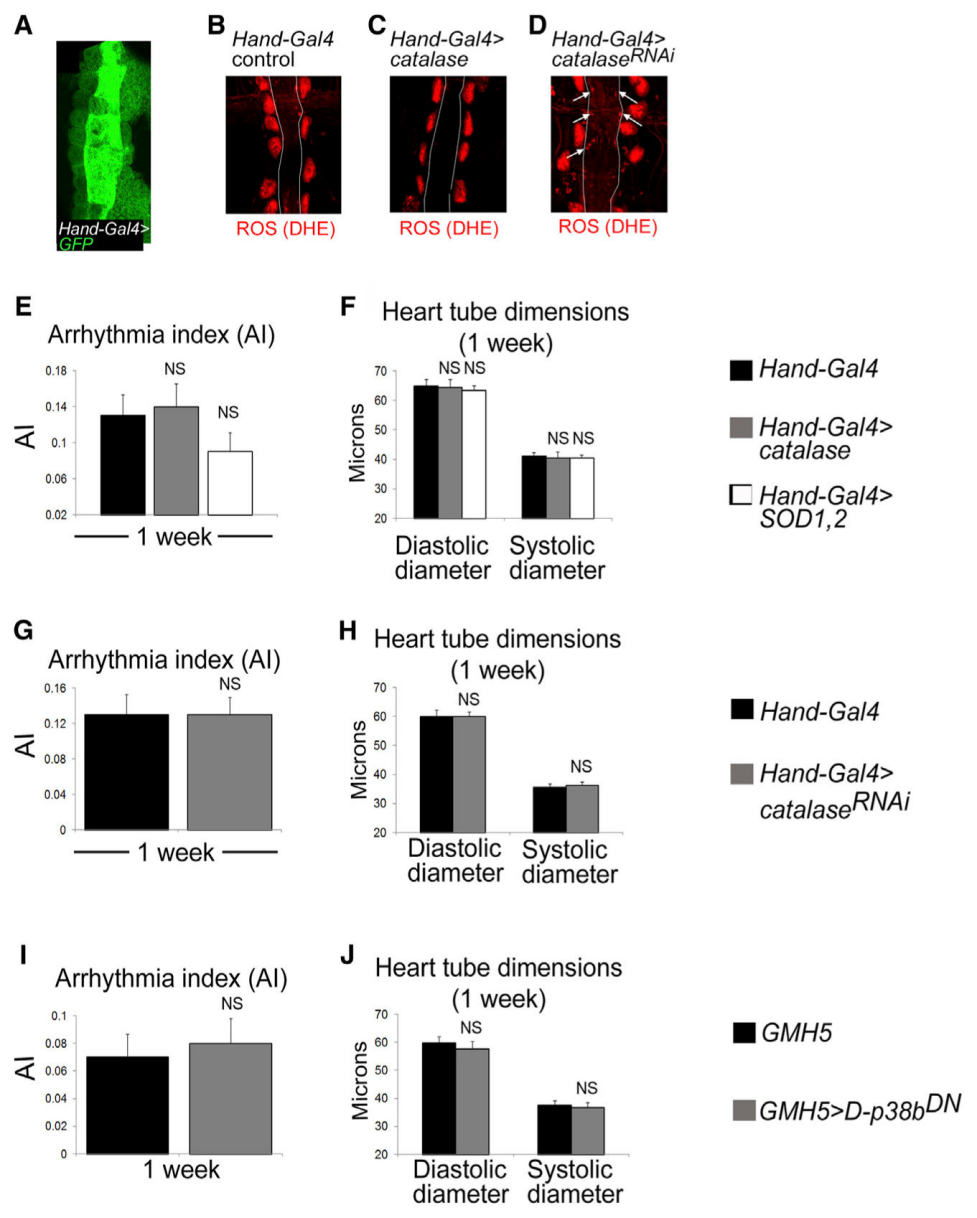
Myocardial-Specific Manipulation of ROS or D-p38 Levels Has No Detectable Effects on Cardiac Function and Morphology (A) Confocal microscopic image of a fixed heart preparation showing myocardial-specific (*Hand-GAL4* driver) *GFP* expression. (B–D) DHE fluorescence in 7- to10-day-old adult control hearts (B), and hearts from adult flies with *Hand-GAL4*-induced *catalase* overexpression (C) or *catalase* RNAi (*catalase^RNAi^*) (D). Arrows indicate increased DHE fluorescence in the myocardium of the *Hand-GAL4 > catalase^RNAi^* hearts (D). Endogenous ROS levels in PCs, as indicated by DHE fluorescence, in hearts with myocardial-specific overexpression of *catalase* (C) or *catalase^RNAi^* (D) are similar to that in control PCs (B). (E–J) AI (E, G, and I) and heart tube dimensions (F, H, and J) in 1-week-old control flies and flies with myocardial-specific overexpression of *catalase* or *SOD1,SOD2* (E and F), *catalase^RNAi^* (G and H), or *D-p38b^DN^* (I and J). All error bars indicate SEM. NS, not significant compared with controls by two-tailed paired t test.

Previous studies have identified a role of H_2_O_2_ serving as paracrine-diffusible mediators under pathological stimulation. For instance, in response to tissue damage in the zebrafish, H_2_O_2_ is produced and secreted from wounded epithelial cells and diffuses into neutrophils to mediate their recruitment to the wound (Niethammer et al., 2009; Yoo et al., 2011). We therefore examined the possibility that physiological ROS might diffuse from PCs into the neighboring CMs and function within the CMs to regulate normal heart performance in *Drosophila*. However, we observed that the myocardium, under normal conditions, displayed undetectable levels of ROS reporter expression, including DHE, *roGFP2-Orp1*, and *GSTD1-GFP* expression (Figures 1B, 1C, 1F, S1A, and S1B). In addition, using the same ROS reporters, we found that modulation of pericardial ROS levels did not induce detectable changes in ROS levels in the CMs (Figures 1D, 1E, and S1C–S1G), a condition that is expected if ROS in the CMs are derived from pericardial ROS. We further reasoned that if the cardiac effects of PC-derived ROS were indeed mediated by ROS directly diffusing into and acting within the neighboring CMs, we would expect that experimental manipulation of ROS levels directly in the CMs to recapitulate the effects in cardiac function or morphology caused by perturbations of peri-cardial ROS concentrations. To test this, we overexpressed *catalase*, *SOD1*, and *SOD*2, or *catalase^RNAi^* using *Hand-GAL4*, which drives expression more strongly in CMs than in PCs (Figure 2A). We found that reducing (Figure 2C) or increasing (Figure 2D) ROS concentrations in the myocardium relative to control (Figure 2B) had no significant effects on any major aspects of heart function, including heart rhythm or heart tube dimensions (Figures 2E–2H). These results therefore argue against the notion that ROS act as diffusible paracrine signaling molecules from PCs into the CMs to modulate normal heart function. Instead, we postulate that ROS signaling controls the production of downstream signals that in turn act on the CMs in a paracrine fashion to regulate their function.

### Perturbation of D-p38 Signaling Phenocopies Hearts with Pericardial Reduction of ROS Levels

Next, we sought to identify the pericardial ROS effectors that impact the CMs and direct their proper function. Several redox-sensitive signaling proteins have been identified, including the mitogen-activated protein kinases (MAPKs) (Griendling et al., 2000). Although activation of MAPKs by excessive ROS is most commonly associated with tissue dysfunction and disease pathogenesis (Giordano, 2005; Griendling et al., 2000), it is possible that MAPKs are also regulated by physiological ROS levels. To examine whether physiological ROS modulate cardiac function by regulating the *Drosophila* MAPK family member D-p38 signaling in PCs, we first examined whether D-*p38* mutant flies incur any cardiac dysfunction. We analyzed flies lacking both the *D-*p38-encoding genes, *D-p38a* and *D-p38b*. At 1 week old, the *D-p38a^−/−^, D-p38b^−/−^* double-mutant flies had abnormal heart function, as characterized by an increased incidence of irregular beats, broader distribution of HPs, and significantly elevated the AI compared with wild-type flies (Figures S5A–S5E). Moreover, the diastolic and systolic diameters were significantly smaller in *D-p38a^−/−^,D-p38b^−/−^* than in wild-type hearts, resulting in a narrower heart tube (Figures S5F–S5H). Single-mutant *D-p38a^−/−^* or *D-p38b^−/−^* hearts exhibited no defects under baseline conditions (Na et al., 2013). These heart abnormalities in the double mutant were reminiscent of the cardiac effects of flies with reduced pericardial ROS levels, suggesting that D-p38a/D-p38b might be involved in ROS signaling in PCs.

**Figure 3:**
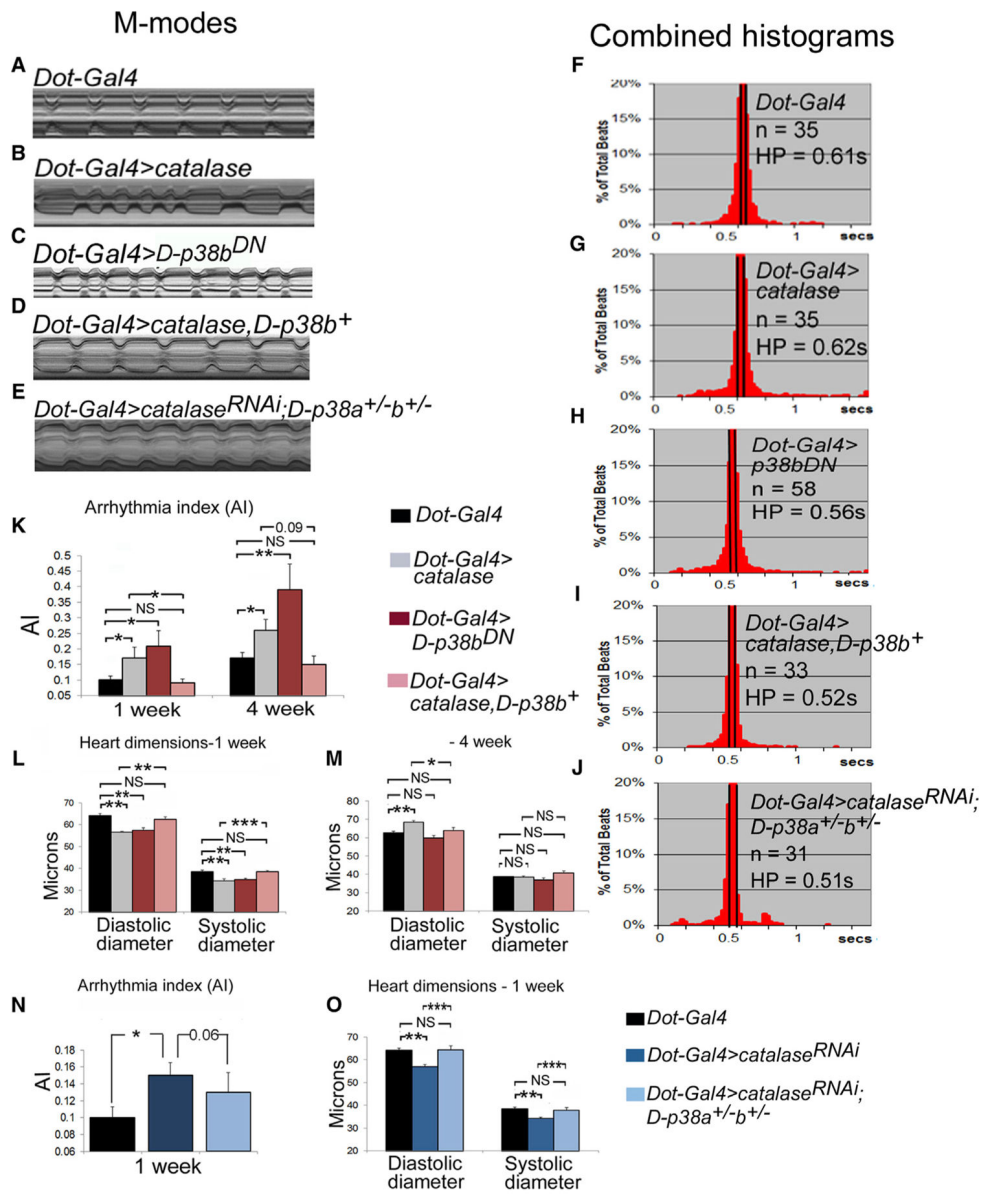
Inhibition of D-p38 Signaling in PCs Elicits Cardiac Dysfunction (A–E) Representative 5 s M-mode traces from hearts of 1-week-old control flies (A), or flies with PC-specific overexpression of *catalase* (B), D-*p38b^DN^* (C), *catalase* and wild-type *D-p38b* (D), or *catalase^RNAi^* and reduced dosage of both *D-p38a* and *D-p38b* (E). (F–J) Combined histograms showing the distribution of the HP for 1-week-old flies. n, number of flies. (K–M) AI (K) and heart dimensions (L and M) in 1- and 4-week-old flies. (N and O) AI (N) and heart dimensions (O) in 1-week-old flies. All error bars indicate SEM. *p < 0.05 and **p < 0.01 compared with *Dot-GAL4* controls by two-tailed paired t test and one-way ANOVA. NS, not significant. See also Figures S5–S7.

Next, to determine if the interference of D-p38 function specifically in PCs also compromises normal heart function, we expressed a dominant-negative form of *D-p38b* (*D-p38b^DN^*) in PCs, in which the MAPK kinase (MAPKK) activation target site was mutated (Adachi-Yamada et al., 1999). Like the *p38a^−/−^p38b^−/−^* double-mutant flies, the pericardial p38b^DN^-expressing (*Dot-GAL4 > p38b^DN^*) flies exhibited cardiac defects that mimicked those of the pericardial ROS-reduced hearts. Specifically, cardiac arrhythmias were elevated in the flies with pericardial overexpression of *D-p38b^DN^* relative to control flies (Figures 3A–3C, 3F–3H, 3K, S2E, S2I, and S2J). Moreover, the *D-p38b^DN^* genotype was also associated with a constriction of the cardiac tube in young (1 week) but not old (4 weeks) flies (Figures 3L, 3M, S2K, and S6A–S6C). Thus, flies with PC-specific reductions in ROS levels and D-p38 MAPK activity exhibited similar cardiac defects, including elevated arrhythmias and heart tube remodeling (compare Figures 1 and 3). In contrast, CM-targeted inhibition of D-p38 activity (*GMH5 > D-p38b^DN^*) had no detectable effects on cardiac function and morphology (Figures 2I and 2J), consistent with that observed with the CM-specific alterations of ROS levels (Figures 2E–2H). These data show that D-p38 signaling in PCs, like that of ROS, is essential for normal cardiac function.

### D-p38 Acts as a Downstream Functional Target of ROS in PCs for Regulating Normal Heart Physiology

Examining whether D-p38 functions as a target of ROS in PCs, we studied the effects of altering ROS levels on D-p38 phosphorylation, a reflection of its activity. We found that suppressing or enhancing ROS concentrations in PCs (*Dot-GAL4>catalase* and *Dot-GAL4 > catalase^RNAi^*) decreased or increased the phosphorylation of D-p38, respectively (Figures S6D–S6F). These results are consistent with the notion that D-p38 activity is controlled by ROS in PCs. We further asked whether D-p38 acts downstream of ROS to mediate paracrine control of normal heart function. If that is the case, we reasoned that the cardiac defects associated with decreased ROS levels should be rescued by PC-specific overexpression of D-p38. Indeed, the cardiac arrhythmia and tube narrowing phenotypes associated with decreased pericardial ROS levels were significantly rescued by overexpressing wild-type D-p38b (*D-p38b^+^*) in PCs (Figures 3D, 3I, 3K, and 3L). Conversely, decreasing the dosage of *D-p38a* and *D-p38b* in flies harboring *Dot-GAL4*-mediated KD of *catalase* partially restored normal heart rhythm and normalized the diastolic and systolic diameters (Figures 3E, 3J, 3N, and 3O). Collectively, these results provide strong evidence that the cardiac defects associated with altered pericardial ROS levels are mediated by D-p38 signaling, thus suggesting that D-p38 is a downstream effector of ROS signaling in PCs.

### A D-MKK3-D-p38 Signaling Axis Mediates the Effects of ROS from PCs to CMs

We next asked whether D-MKK3 might function as the MAPKK upstream of D-p38 in this ROS signaling pathway. We found that PC-specific KD of D-MKK3 (*Dot-GAL4 > D-MKK3^RNAi^*) significantly increased the frequency of arrhythmias and caused age-dependent heart tube remodeling (Figures S7A–S7G). The similarity between these cardiac defects and those observed upon PC-specific reduction of ROS and D-p38 levels is thus consistent with the idea that D-MKK3 mediates the ROS-induced activation of D-p38. In support, we found that altered expression of D-MKK3 resulted in correspondingly changed phosphorylation of nuclear D-p38 (Figures S7H–S7J). These data place D-MKK3 in the pericardial ROS-D-p38 signaling cascade that critically modulates cardiac function in a paracrine fashion.

### ROS-D-p38 Signaling in PCs during Development Is Important in Establishing Adult Normal Heart Function

**Figure 4:**
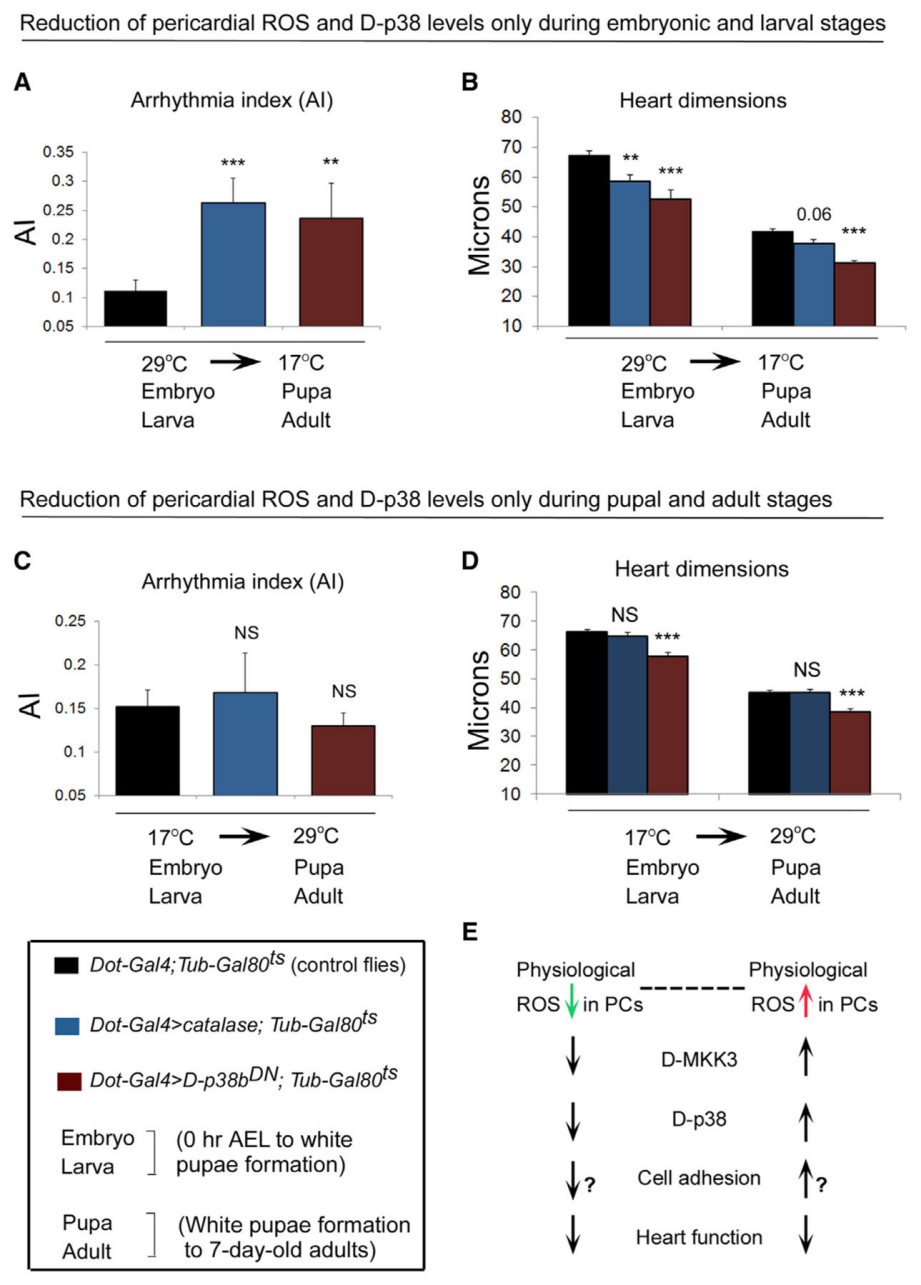
ROS-D-p38 Signaling in PCs during Development Is Important in Establishing Adult Normal Heart Function (A and B) AI (A) and heart tube dimensions (B) in 1-week-old control flies (black bars) and flies with PC-specific overexpression of *catalase* (blue bars) or *D-p38b^DN^* (brown bars) only during the embryonic and larval phases. (C and D) AI (C) and heart tube dimensions (D) in 1-week-old control flies (black bars) and flies with PC-specific overexpression of *catalase* (blue bars) or *D-p38b^DN^* (brown bars) only during the pupal and adult phases. All error bars indicate SEM. **p < 0.01 and ***p < 0.001 compared with *Dot-GAL4* controls by two-tailed paired t test. NS, not significant. (E) Model proposing the mechanism by which nonphysiologically high or low levels of ROS in PCs could adversely affect cardiac function. The pericardial-intrinsic ROS-D-MKK3-D-p38 signaling could modulate the expression of pericardial adhesion molecules that are in contact with adjacent CMs, thereby altering cell-cell adhesion between PCs and the CMs and, consequently, CM function.

Physiological ROS were found to be present in PCs during larval in addition to adult stages (Figures 1B, 1C, S1A, and S1B), implying that ROS could play a role in establishing and modulating adult heart performance. Because the pericardial *Dot-GAL4* and *Sns-GCN-GAL4* drivers are also active during development (Kimbrell et al., 2002; Zhuang et al., 2009), ROS and D-p38 manipulation in embryonic and larval PCs may also influence adult heart function. To distinguish between the developmental versus adult effects of ROS-D-p38 signaling in PCs on heart function, we manipulated catalase and D-p38 levels either during the embryonic and larval stages only, or during the pupal and adult stages only, using the *Gal80^ts^*system. Gal80^ts^ is a temperature-sensitive, reversible inhibitor of GAL4 (Osterwalder et al., 2001; Roman et al., 2001). At the nonpermissive temperature (17°C), Gal80^ts^ binds GAL4 to inhibit GAL4-mediated transcriptional activation, whereas at the permissive temperature (29°C), it dissociates from GAL4 to permit GAL4-mediated gene expression. To induce a decrease in pericardial ROS or D-p38 levels during the embryonic and larval stages only, flies harboring *Dot-GAL4* and a *Gal80^ts^* construct driven by the *Tubulin* promoter (*Dot-GAL4;Tub-Gal80^ts^*) were crossed to either *UAS-catalase* or *UAS-D-p38b^DN^* (*Dot-GAL4 > catalase;Tub-Gal80^ts^* and *Dot-GAL4 > D-p38b^DN^;Tub-Gal80^ts^*, Figure 4). The progenies were reared at 29°C during embryonic and larval stages until white pupae formation, which was followed by a temperature shift to 17°C until 7 days of adulthood, and heart function was analyzed (Figures 4A and 4B). Reciprocally, to induce a decrease in pericardial ROS or D-p38 levels during the pupal and adult stages only, flies were reared at 17°C up to white pupae formation followed by a temperature shift to 29°C until 7 days of adulthood, followed by heart function analysis (Figures 4C and 4D). Interestingly, we found that overexpression of catalase or D-p38b^DN^ in PCs during the embryonic and larval stages only resulted in significant cardiac arrhythmias compared to the controls (Figure 4A). Moreover, the perturbation of pericardial ROS-D-p38 signaling during the embryonic and larval stages only leads in addition to a significant thinning of the cardiac tube (Figure 4B). In contrast, interference of D-p38 activity in PCs during the pupal and adult stages only was associated with a significant narrowing of the cardiac tube (Figure 4D) but does not affect the AI (Figure 4C). Taken together, these data indicate that the level of ROS-D-p38 signaling in PCs is critical for normal adult heart function, in particular during development.

## DISCUSSION

The consensus view of ROS as signaling molecules is that ROS act in a cell-autonomous manner to induce physiological or pathophysiological responses in the ROS-generating cell. Paracrine roles of ROS signaling have more recently been reported to occur under pathological conditions, such as upon tissue wounding whereby ROS diffuse from their site of production to act on surrounding cells (Niethammer et al., 2009; Yoo et al., 2011). Our study demonstrates that ROS that are naturally present in the nonmyocytic PCs can mediate paracrine signaling to the adjacent CMs under physiological conditions that is critical for proper cardiac function. Furthermore, we showed that this occurred not via the extracellular distribution of ROS from PCs into the CMs, but rather via ROS inducing the activation of downstream D-MKK3-D-p38 signaling in PCs that then causes the CMs to maintain their normal function.

It is now recognized that the constitutive generation of moderate amounts of ROS is important for normal physiological processes (Owusu-Ansah and Banerjee, 2009). In the heart, moderate and controlled levels of ROS could promote myocyte growth (Sugden and Clerk, 2006), regulate vascular smooth muscle tone (Suvorava and Kojda, 2009), and act as protective signaling elements during the preischemic phase (Kevin et al., 2005). However, the precise mechanisms by which ROS maintain cardiac homeostasis have yet to be established, particularly the ROS-mediated paracrine signaling mechanisms that are crucial for proper heart function. Cell-to-cell interactions are typically mediated by soluble factors, cell-cell adhesion complexes, and, indirectly, by the surrounding ECM. In the vascular cells such as endothelial cells, expression of several adhesion molecules, including vascular cell adhesion molecule 1 (VCAM-1) and intracellular adhesion molecule 1 (ICAM-1), is ROS dependent (Taniyama and Griendling, 2003). Furthermore, ROS have been found to modulate the activity and expression levels of the matrix metalloproteinases in vascular smooth muscle cells that contribute to physiological and pathological vascular remodeling (Fu et al., 2001; Grote et al., 2003). Thus, it will be interesting to determine the potential roles of various PC adhesion molecules and the surrounding ECM proteins as downstream targets of the ROS-D-MKK3-D-p38 signaling axis in PCs that mediate their paracrine effects on the CMs (Figure 4E). Some of the potential candidates could include septate junction (SJ) proteins such as Coracle and Neurexin IV, which localize to the plasma membranes of PCs and CMs in the *Drosophila* embryo and mediate PC-CM adhesion and proper heart function (Yi et al., 2008), as well as the cardiac ECM protein Pericardin, which is crucial for heart morphogenesis and cardiac cell-to-PC adhesion (Chartier et al., 2002; Yi et al., 2008). The insights derived from the delineation of this physiological ROS-mediated signaling mechanism between PCs and CMs could lead to a more complete understanding of the functional interactions between cardiac myocytes and nonmyocytes, as well as of cell-to-cell communications in other tissues.

## EXPERIMENTAL PROCEDURES

### Fly Stocks

UAS-catalase, UAS-SOD1, UAS-SOD2, UAS-p38b^+^, UAS-DMKK3^+^, UAS-coracle^+^, Tubulin-GAL4, Armadillo-GAL4 (Arm-GAL4), and Tubulin-Gal80^ts^ were from the Bloomington Stock Center. UAS-catalase^RNAi^, UAS-SOD1^RNAi^, UAS-SOD2^RNAi^, UAS-DMKK3^RNAi^, and UAS-coracle^RNAi^ were from the Vienna *Drosophila* RNAi Center. D-p38a^13^ and D-p38b^156A^ were as previously described (Chen et al., 2010). UAS-p38b^DN^ (Adachi-Yamada et al., 1999) was a kind gift from T. Adachi-Yamada at Kobe University, Japan. Dot-GAL4 (insertion 11C (c2)) (Kimbrell et al., 2002) was previously generated in D.A. Kimbrell’s laboratory at the University of California, Davis. GMH5 was as previously described by Wessells et al. (2004). Hand-GAL4 was a kind gift from A. Paululat (University of Osnabruek, Germany).

### Temperature Shift Assays

The overexpression of *UAS* transgene was induced only during the embryonic and larval phases (from ~0 hr after egg laying [AEL] to white pupae formation) or only during the pupal and adult phases (from white pupae formation to 7-day-old adulthood). To induce transgene overexpression only during the embryonic and larval phases, fertilized eggs were collected at room temperature (RT) on standard food vials, after which vials were transferred to 29°C. Larvae were maintained at 29°C until the onset of puparium formation (white pupae). Upon white pupae formation, vials were transferred to 17°C for culture until the eclosion of adult flies. Adult flies were continued to be raised at 17°C for about 7 days before being analyzed for their cardiac function (at RT). To induce *UAS*-transgene overexpression only during the pupal and adult phases, the same procedures were carried out except that the temperatures for fly rearing were reversed.

### ROS Detection

ROS detection with DHE dye (Molecular Probes, Invitrogen) was performed using a published method (Owusu-Ansah and Banerjee, 2009), with minor modifications. In brief, adult fly hearts were dissected and cleaned in freshly prepared PBS and removed from the cuticle. Hearts were incubated with 30 μM DHE (freshly reconstituted in anhydrous DMSO and diluted in PBS) for 7–10 min at RT in the dark, washed three times with PBS for 5 min each in the dark, and then fixed for 5 min with 7% paraformaldehyde (PFA). Hearts were mounted in ProLong Gold antifade reagent (Invitrogen) and examined under a laser confocal microscope (Zeiss). The endogenous redox state in PCs and CMs was monitored using a genetically encoded fluorescent redox reporter, cyto-roGFP2-Orp1 (Albrecht et al., 2011). Orp1-mediated oxidation induces a conformational change in the linked roGFP2 variant that decreases its fluorescence. Detection of ROS (H_2_O_2_) with cyto-roGFP2-Orp1 in the hearts was performed as previously described (Albrecht et al., 2011). In brief, adult fly hearts were dissected and incubated for 10 min at RT in freshly prepared PBS containing 20 mM N-ethylmaleimide. Hearts were rinsed once with PBS, fixed with 4% PFA for 15 min at RT, and then washed twice with PBS for 10 min. Hearts were mounted overnight in ProLong Gold antifade reagent and examined under a laser confocal microscope (Zeiss) with excitation at 488 and 405 nm.

### Immunodetection Reagents

The following reagents were used for immunostaining: rabbit monoclonal anti-phospho-p38 (Thr180/Tyr182, clone 3D7; Cell Signaling Technology) at 1:100; fluorescein-labeled phalloidin (Invitrogen) at 1:50; and mouse monoclonal anti-actinin (Developmental Studies Hybridoma Bank) at 1:50.

### Immunostaining

Third-instar wandering-stage larvae and adult female flies (7–10 days old) were collected and dissected in PBS. Hearts were fixed in a solution comprising picric acid/glacial acetic acid/formaldehyde in a ratio of 15:1:5 for 15 min at RT. After washing in PBS plus 0.1% Triton X-100 (PBT), the fixed hearts were incubated overnight at 4°C with primary antibodies diluted in PBT. Hearts were then washed with PBT and incubated for 2 hr at RT with the appropriate fluorescence-conjugated secondary antibodies (Jackson ImmunoResearch) diluted in PBT. Hearts were then washed again with PBT and mounted in ProLong Gold antifade reagent. Samples were examined under an epifluorescence-equipped (Olympus) or laser confocal (Zeiss) microscope.

### Fly Heartbeat Analysis

Cardiac contractility measurements on semi-intact preparations of fly hearts were performed as described previously by Fink et al. (2009). High-speed 30 s movies were recorded at a rate of >150 frames per second using a Hamamatsu CCD camera on a Nikon 80i upright microscope with a 10× dipping immersion lens (see Fink et al. [2009] for further details). The images were processed using SimplePCI software (Compix). M-modes and quantitative data were generated using a MATLAB-based image analysis program (Fink et al., 2009). To generate the M-mode figures, a single pixel-wide column was selected from the most posterior portion of the adult heart at the abdominal A3 segment that encompassed both edges of the heart tube. The corresponding columns were cut from all movie frames and aligned horizontally according to time. HPs or heartbeat lengths were defined as the time between the ends of two consecutive diastolic intervals. The AI was defined as the SD of all recorded HPs for an individual fly, normalized to the median HP to compensate for variability between flies (Ocorr et al., 2007). Diastolic and systolic diameters represent the relaxed and contracted state of the heart tube, respectively. Measurements were made in the exact same location in abdominal segment A3.

## Supplementary Material
