# Supplementary material for: ROS Regulate Cardiac Function via a Distinct Paracrine Mechanism
Source: Cell Rep. Author manuscript; Available in PMC 2014 Sep 15. (PMC4164050; doi:10.1016/j.celrep.2014.02.029)
Supplement: Supplementary file 1 [file NIHMS625674-supplement-supplement_1.pdf]

signaling elements during the preischemic phase (Kevin et al., 2005). However, the precise mechanisms by which ROS maintain cardiac homeostasis have yet to be established, particularly the ROS-mediated paracrine signaling mechanisms that are crucial for proper heart function. Cell-to-cell interactions are typically mediated by soluble factors, cell-cell adhesion complexes, and, indirectly, by the surrounding ECM. In the vascular cells such as endothelial cells, expression of several adhesion molecules, including vascular cell adhesion molecule 1 (VCAM-1) and intracellular adhesion molecule 1 (ICAM-1), is ROS dependent (Taniyama and Griendling, 2003). Furthermore, ROS have been found to modulate the activity and expression levels of the matrix metalloproteinases in vascular smooth muscle cells that contribute to physiological and pathological vascular remodeling (Fu et al., 2001; Grote et al., 2003). Thus, it will be interesting to determine the potential roles of various PC adhesion molecules and the surrounding ECM proteins as downstream targets of the ROS-D-MKK3-D-p38 signaling axis in PCs that mediate their paracrine effects on the CMs (Figure 4E). Some of the potential candidates could include septate junction (SJ) proteins such as Coracle and Neurexin IV, which localize to the plasma membranes of PCs and CMs in the *Drosophila* embryo and mediate PC-CM adhesion and proper heart function (Yi et al., 2008), as well as the cardiac ECM protein Pericardin, which is crucial for heart morphogenesis and cardiac cell-to-PC adhesion (Chartier et al., 2002; Yi et al., 2008). The insights derived from the delineation of this physiological ROS-mediated signaling mechanism between PCs and CMs could lead to a more complete understanding of the functional interactions between cardiac myocytes and nonmyocytes, as well as of cell-to-cell communications in other tissues.

EXPERIMENTAL PROCEDURES

Fly Stocks

UAS-catalase, UAS-SOD1, UAS-SOD2, UAS-p38b$^+$, UAS-DMKK3$^+$, UAS-coracle$^+$, Tubulin-GAL4, Armadillo-GAL4 (Arm-GAL4), and Tubulin-Gal80^{ts} were from the Bloomington Stock Center. UAS-catalase^{RNAi}, UAS-SOD1^{RNAi}, UAS-SOD2^{RNAi}, UAS-DMKK3^{RNAi}, and UAS-coracle^{RNAi} were from the Vienna *Drosophila* RNAi Center. D-p38a^{13} and D-p38b^{156A} were as previously described (Chen et al., 2010).UAS-p38b^{DN} (Adachi-Yamada et al., 1999) was a kind gift from T. Adachi-Yamada at Kobe University, Japan. Dot-GAL4 (insertion 11C (c2)) (Kimbrell et al., 2002) was previously generated in D.A. Kimbrell's laboratory at the University of California, Davis. GMH5 was as previously described by Wessells et al. (2004). Hand-GAL4 was a kind gift from A. Paululat (University of Osnabruek, Germany).

Temperature Shift Assays

The overexpression of *UAS* transgene was induced only during the embryonic and larval phases (from ~0 hr after egg laying [AEL] to white pupae formation) or only during the pupal and adult phases (from white pupae formation to 7-day-old adulthood). To induce transgene overexpression only during the embryonic and larval phases, fertilized eggs were collected at room temperature (RT) on standard food vials, after which vials were transferred to 29°C. Larvae were maintained at 29°C until the onset of puparium formation (white pupae). Upon white pupae formation, vials were transferred to 17°C for culture until the eclosion of adult flies. Adult flies were continued to be raised at 17°C for about 7 days before being analyzed for their cardiac function (at RT). To induce *UAS*-transgene overexpression only during the pupal and adult phases, the same procedures were carried out except that the temperatures for fly rearing were reversed.

ROS Detection

ROS detection with DHE dye (Molecular Probes, Invitrogen) was performed using a published method (Owusu-Ansah and Banerjee, 2009), with minor modifications. In brief, adult fly hearts were dissected and cleaned in freshly prepared PBS and removed from the cuticle. Hearts were incubated with 30 μM DHE (freshly reconstituted in anhydrous DMSO and diluted in PBS) for 7–10 min at RT in the dark, washed three times with PBS for 5 min each in the dark, and then fixed for 5 min with 7% paraformaldehyde (PFA). Hearts were mounted in ProLong Gold antifade reagent (Invitrogen) and examined under a laser confocal microscope (Zeiss). The endogenous redox state in PCs and CMs was monitored using a genetically encoded fluorescent redox reporter, cyto-roGFP2-Orp1 (Albrecht et al., 2011).Orp1-mediated oxidation induces a conformational change in the linked roGFP2 variant that decreases its fluorescence. Detection of ROS (H_2O_2) with cyto-roGFP2-Orp1 in the hearts was performed as previously described (Albrecht et al., 2011). In brief, adult fly hearts were dissected and incubated for 10 min at RT in freshly prepared PBS containing 20 mM N-ethylmaleimide. Hearts were rinsed once with PBS, fixed with 4% PFA for 15 min at RT, and then washed twice with PBS for 10 min. Hearts were mounted overnight in ProLong Gold antifade reagent and examined under a laser confocal microscope (Zeiss) with excitation at 488 and 405 nm.

Immunodetection Reagents

The following reagents were used for immunostaining: rabbit monoclonal anti-phospho-p38 (Thr180/Tyr182, clone 3D7; Cell Signaling Technology) at 1:100; fluorescein-labeled phalloidin (Invitrogen) at 1:50; and mouse monoclonal anti-actinin (Developmental Studies Hybridoma Bank) at 1:50.

Immunostaining

Third-instar wandering-stage larvae and adult female flies (7–10 days old) were collected and dissected in PBS. Hearts were fixed in a solution comprising picric acid/glacial acetic acid/formaldehyde in a ratio of 15:1:5 for 15 min at RT. After washing in PBS plus 0.1% Triton X-100 (PBT), the fixed hearts were incubated overnight at 4°C with primary antibodies diluted in PBT. Hearts were then washed with PBT and incubated for 2 hr at RT with the appropriate fluorescence-conjugated secondary antibodies (Jackson ImmunoResearch) diluted in PBT. Hearts were then washed again with PBT and mounted in ProLong Gold antifade reagent. Samples were examined under an epifluorescence-equipped (Olympus) or laser confocal (Zeiss) microscope.

Fly Heartbeat Analysis

Cardiac contractility measurements on semi-intact preparations of fly hearts were performed as described previously by Fink et al. (2009). High-speed 30 s movies were recorded at a rate of >150 frames per second using a Hamamatsu CCD camera on a Nikon 80i upright microscope with a 10× dipping immersion lens (see Fink et al. [2009] for further details). The images were processed using SimplePCI software (Compix). M-modes and quantitative data were generated using a MATLAB-based image analysis program (Fink et al., 2009). To generate the M-mode figures, a single pixel-wide column was selected from the most posterior portion of the adult heart at the abdominal A3 segment that encompassed both edges of the heart tube. The corresponding columns were cut from all movie frames and aligned horizontally according to time. HPs or heartbeat lengths were defined as the time between the ends of two consecutive diastolic intervals. The AI was defined as the SD of all recorded HPs for an individual fly, normalized to the median HP to compensate for variability between flies (Ocorr et al., 2007). Diastolic and systolic diameters represent the relaxed and contracted state of the heart tube, respectively. Measurements were made in the exact same location in abdominal segment A3.

SUPPLEMENTAL INFORMATION

Supplemental Information includes seven figures and can be found with this article online at http://dx.doi.org/10.1016/j.celrep.2014.02.029.

ACKNOWLEDGMENTS

We thank our colleagues S. Lipton, M. Mercola, P.L. Puri, and Z. Ronai at SBMRI and D. Capra, L. Thompson, L. Szweda, X.H. Sun, and J. Knight at OMRF for comments on previous versions of the manuscript, and Gary Struhl at Columbia University for critical comments on the current manuscript. We acknowledge the Imaging Core Facility at OMRF for excellent technical assistance. We are grateful to T. Adachi-Yamada (Kobe University), R. Fehon (University of Chicago), Bloomington Stock Center, DGRC (Japan), VDRC (Austria), and DSHB (Iowa) for fly strains and reagents. This work was supported by fellowships from the American Heart Association (0825276F and 10POST4140064 to H.-Y.L.) and grants from the National Institutes of Health (1P20GM103636 to H.-Y.L. and R01HL54732, P01HL098053, and P01AG033456 to R.B.).

Received: January 7, 2014
Revised: February 10, 2014
Accepted: February 20, 2014
Published: March 20, 2014

REFERENCES

Adachi-Yamada, T., Nakamura, M., Irie, K., Tomoyasu, Y., Sano, Y., Mori, E., Goto, S., Ueno, N., Nishida, Y., and Matsumoto, K. (1999). p38 mitogen-activated protein kinase can be involved in transforming growth factor beta superfamily signal transduction in *Drosophila* wing morphogenesis. Mol. Cell. Biol. *19*, 2322–2329.

Albrecht, S.C., Barata, A.G., Grosshans, J., Teleman, A.A., and Dick, T.P. (2011). In vivo mapping of hydrogen peroxide and oxidized glutathione reveals chemical and regional specificity of redox homeostasis. Cell Metab. *14*, 819–829.

Brade, T., Kumar, S., Cunningham, T.J., Chatzi, C., Zhao, X., Cavallero, S., Li, P., Sucov, H.M., Ruiz-Lozano, P., and Duester, G. (2011). Retinoic acid stimulates myocardial expansion by induction of hepatic erythropoietin which activates epicardial Igf2. Development *138*, 139–148.

Brand, A.H., and Perrimon, N. (1993). Targeted gene expression as a means of altering cell fates and generating dominant phenotypes. Development *118*, 401–415.

Buechling, T., Akasaka, T., Vogler, G., Ruiz-Lozano, P., Ocorr, K., and Bodmer, R. (2009). Non-autonomous modulation of heart rhythm, contractility and morphology in adult fruit flies. Dev. Biol. *328*, 483–492.

Chartier, A., Zaffran, S., Astier, M., Sémériva, M., and Gratecos, D. (2002). Pericardin, a *Drosophila* type IV collagen-like protein is involved in the morphogenesis and maintenance of the heart epithelium during dorsal ectoderm closure. Development *129*, 3241–3253.

Chen, J., Xie, C., Tian, L., Hong, L., Wu, X., and Han, J. (2010). Participation of the p38 pathway in *Drosophila* host defense against pathogenic bacteria and fungi. Proc. Natl. Acad. Sci. USA *107*, 20774–20779.

Covarrubias, L., Hernández-García, D., Schnabel, D., Salas-Vidal, E., and Castro-Obregón, S. (2008). Function of reactive oxygen species during animal development: passive or active? Dev. Biol. *320*, 1–11.

Dröge, W. (2002). Free radicals in the physiological control of cell function. Physiol. Rev. *82*, 47–95.

Fink, M., Callol-Massot, C., Chu, A., Ruiz-Lozano, P., Izpisua Belmonte, J.C., Giles, W., Bodmer, R., and Ocorr, K. (2009). A new method for detection and quantification of heartbeat parameters in *Drosophila*, zebrafish, and embryonic mouse hearts. Biotechniques *46*, 101–113.

Fu, X., Kassim, S.Y., Parks, W.C., and Heinecke, J.W. (2001). Hypochlorous acid oxygenates the cysteine switch domain of pro-matrilysin (MMP-7). A mechanism for matrix metalloproteinase activation and atherosclerotic plaque rupture by myeloperoxidase. J. Biol. Chem. *276*, 41279–41287.

Fujioka, M., Wessells, R.J., Han, Z., Liu, J., Fitzgerald, K., Yusibova, G.L., Zamora, M., Ruiz-Lozano, P., Bodmer, R., and Jaynes, J.B. (2005). Embryonic even skipped-dependent muscle and heart cell fates are required for normal adult activity, heart function, and lifespan. Circ. Res. *97*, 1108–1114.

Giordano, F.J. (2005). Oxygen, oxidative stress, hypoxia, and heart failure. J. Clin. Invest. *115*, 500–508.

Griendling, K.K., Sorescu, D., Lassègue, B., and Ushio-Fukai, M. (2000). Modulation of protein kinase activity and gene expression by reactive oxygen species and their role in vascular physiology and pathophysiology. Arterioscler. Thromb. Vasc. Biol. *20*, 2175–2183.

Grote, K., Flach, I., Luchtefeld, M., Akin, E., Holland, S.M., Drexler, H., and Schieffer, B. (2003). Mechanical stretch enhances mRNA expression and proenzyme release of matrix metalloproteinase-2 (MMP-2) via NAD(P)H oxidase-derived reactive oxygen species. Circ. Res. *92*, e80–e86.

Kevin, L.G., Novalija, E., and Stowe, D.F. (2005). Reactive oxygen species as mediators of cardiac injury and protection: the relevance to anesthesia practice. Anesth. Analg. *101*, 1275–1287.

Kimbrell, D.A., Hice, C., Bolduc, C., Kleinhesselink, K., and Beckingham, K. (2002). The Dorothy enhancer has Tinman binding sites and drives hopscotch-induced tumor formation. Genesis *34*, 23–28.

Love, N.R., Chen, Y., Ishibashi, S., Kritsiligkou, P., Lea, R., Koh, Y., Gallop, J.L., Dorey, K., and Amaya, E. (2013). Amputation-induced reactive oxygen species are required for successful *Xenopus* tadpole tail regeneration. Nat. Cell Biol. *15*, 222–228.

Merki, E., Zamora, M., Raya, A., Kawakami, Y., Wang, J., Zhang, X., Burch, J., Kubalak, S.W., Kaliman, P., Izpisua Belmonte, J.C., et al. (2005). Epicardial retinoid X receptor alpha is required for myocardial growth and coronary artery formation. Proc. Natl. Acad. Sci. USA *102*, 18455–18460.

Miller, A.F. (2012). Superoxide dismutases: ancient enzymes and new insights. FEBS Lett. *586*, 585–595.

Na, J., Musselman, L.P., Pendse, J., Baranski, T.J., Bodmer, R., Ocorr, K., and Cagan, R. (2013). A *Drosophila* model of high sugar diet-induced cardiomyopathy. PLoS Genet. *9*, e1003175.

Niethammer, P., Grabher, C., Look, A.T., and Mitchison, T.J. (2009). A tissue-scale gradient of hydrogen peroxide mediates rapid wound detection in zebrafish. Nature *459*, 996–999.

Ocorr, K., Reeves, N.L., Wessells, R.J., Fink, M., Chen, H.S., Akasaka, T., Yasuda, S., Metzger, J.M., Giles, W., Posakony, J.W., and Bodmer, R. (2007). KCNQ potassium channel mutations cause cardiac arrhythmias in *Drosophila* that mimic the effects of aging. Proc. Natl. Acad. Sci. USA *104*, 3943–3948.

Ocorr, K., Fink, M., Cammarato, A., Bernstein, S., and Bodmer, R. (2009). Semi-automated Optical Heartbeat Analysis of small hearts. J. Vis. Exp. *31*, 1435.

Osterwalder, T., Yoon, K.S., White, B.H., and Keshishian, H. (2001). A conditional tissue-specific transgene expression system using inducible GAL4. Proc. Natl. Acad. Sci. USA *98*, 12596–12601.

Owusu-Ansah, E., and Banerjee, U. (2009). Reactive oxygen species prime *Drosophila* haematopoietic progenitors for differentiation. Nature *461*, 537–541.

Roman, G., Endo, K., Zong, L., and Davis, R.L. (2001). P[Switch], a system for spatial and temporal control of gene expression in *Drosophila melanogaster*. Proc. Natl. Acad. Sci. USA *98*, 12602–12607.

Sawicki, R., Singh, S.P., Mondal, A.K., Benes, H., and Zimniak, P. (2003). Cloning, expression and biochemical characterization of one Epsilon-class (GST-3) and ten Delta-class (GST-1) glutathione S-transferases from *Drosophila melanogaster*, and identification of additional nine members of the Epsilon class. Biochem. J. *370*, 661–669.

Sugden, P.H., and Clerk, A. (2006). Oxidative stress and growth-regulating intracellular signaling pathways in cardiac myocytes. Antioxid. Redox Signal. *8*, 2111–2124.

Suvorava, T., and Kojda, G. (2009). Reactive oxygen species as cardiovascular mediators: lessons from endothelial-specific protein overexpression mouse models. Biochim. Biophys. Acta *1787*, 802–810.

Sykiotis, G.P., and Bohmann, D. (2008). Keap1/Nrf2 signaling regulates oxidative stress tolerance and lifespan in *Drosophila*. Dev. Cell *14*, 76–85.

Taniyama, Y., and Griendling, K.K. (2003). Reactive oxygen species in the vasculature: molecular and cellular mechanisms. Hypertension *42*, 1075–1081.

Thannickal, V.J., and Fanburg, B.L. (2000). Reactive oxygen species in cell signaling. Am. J. Physiol. Lung Cell. Mol. Physiol. *279*, L1005–L1028.

Tian, Y., and Morrisey, E.E. (2012). Importance of myocyte-nonmyocyte interactions in cardiac development and disease. Circ. Res. *110*, 1023–1034.

Tirziu, D., Giordano, F.J., and Simons, M. (2010). Cell communications in the heart. Circulation *122*, 928–937.

Wessells, R.J., Fitzgerald, E., Cypser, J.R., Tatar, M., and Bodmer, R. (2004). Insulin regulation of heart function in aging fruit flies. Nat. Genet. *36*, 1275–1281.

Wu, S.C., Liao, C.W., Pan, R.L., and Juang, J.L. (2012). Infection-induced intestinal oxidative stress triggers organ-to-organ immunological communication in *Drosophila*. Cell Host Microbe *11*, 410–417.

Yi, P., Johnson, A.N., Han, Z., Wu, J., and Olson, E.N. (2008). Heterotrimeric G proteins regulate a noncanonical function of septate junction proteins to maintain cardiac integrity in *Drosophila*. Dev. Cell *15*, 704–713.

Yoo, S.K., Starnes, T.W., Deng, Q., and Huttenlocher, A. (2011). Lyn is a redox sensor that mediates leukocyte wound attraction in vivo. Nature *480*, 109–112.

Zámocký, M., Gasselhuber, B., Furtmüller, P.G., and Obinger, C. (2012). Molecular evolution of hydrogen peroxide degrading enzymes. Arch. Biochem. Biophys. *525*, 131–144.

Zhuang, S., Shao, H., Guo, F., Trimble, R., Pearce, E., and Abmayr, S.M. (2009). Sns and Kirre, the *Drosophila* orthologs of Nephrin and Neph1, direct adhesion, fusion and formation of a slit diaphragm-like structure in insect nephrocytes. Development *136*, 2335–2344.

ROS regulate cardiac function in *Drosophila*

via a novel paracrine mechanism

Hui-Ying Lim[1,2,*], Weidong Wang[3], Jianming Chen[4], Karen Ocorr[1], Rolf Bodmer[1,*]

Figure S1

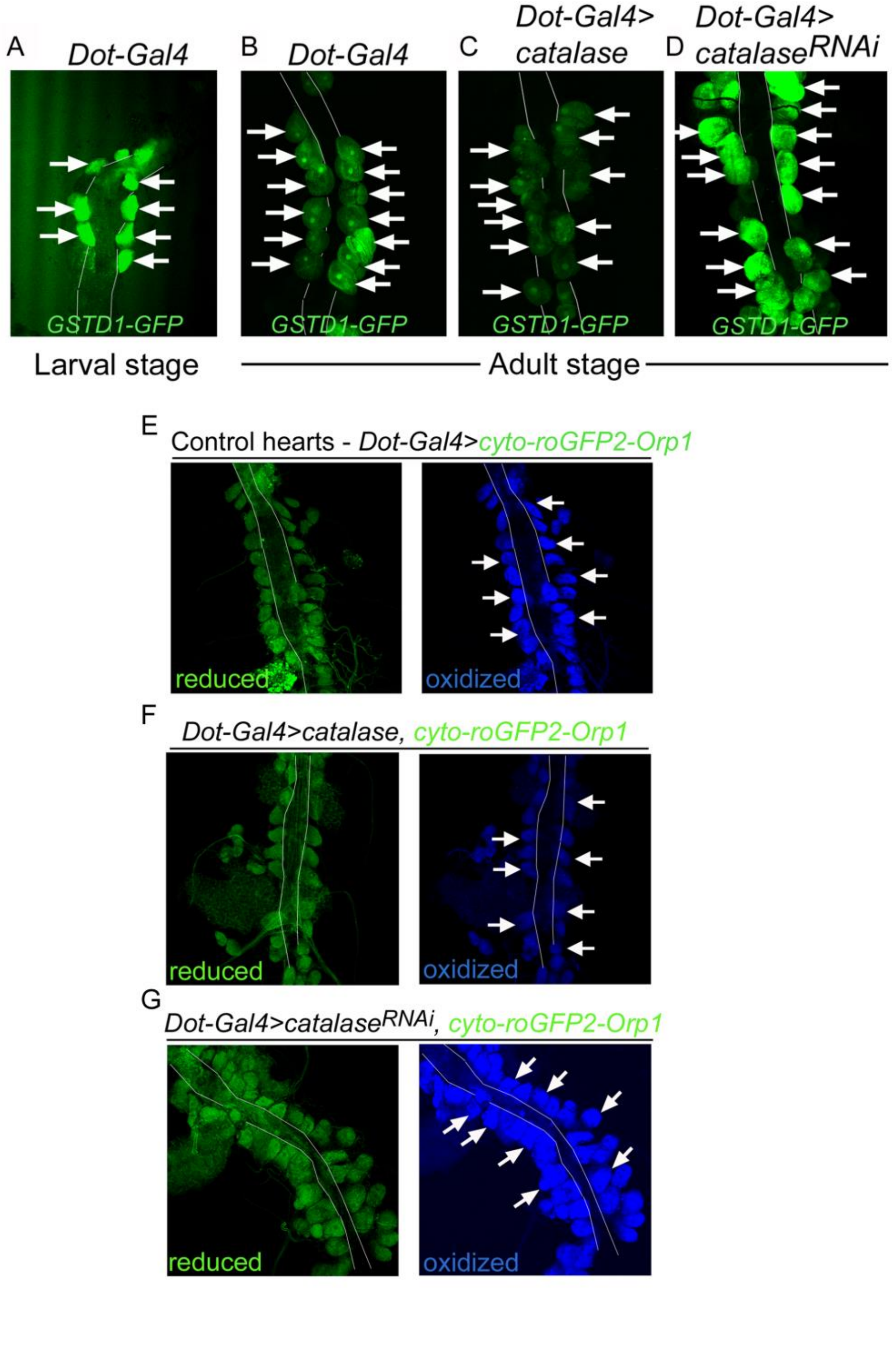
A
Dot-Gal4
B
Dot-Gal4
C
Dot-Gal4>
catalase
D
Dot-Gal4>
catalaseRNAi
GSTD1-GFP
GSTD1-GFP
GSTD1-GFP
GSTD1-GFP
Larval stage
Adult stage
E
Control hearts - Dot-Gal4>cyto-roGFP2-Orp1
reduced
oxidized
F
Dot-Gal4>catalase, cyto-roGFP2-Orp1
reduced
oxidized
G
Dot-Gal4>catalaseRNAi, cyto-roGFP2-Orp1
reduced
oxidized

Supplemental Figure 1. Endogenous oxidation of a genetically-encoded redox probe in PCs, Related to Figure 1.

(A-D) *GSTD1* promoter-driven GFP expression (*GSTD1-GFP*) in control third-instar larval heart (A), adult control heart (B), or hearts with PC-specific (*Dot-Gal4* driver) overexpression of *catalase* (C) or *catalase*^{RNAi} (D). The dotted lines outline the myocardial tubes.

(E-G) The oxidation status (reduced, green; oxidized, blue) of PCs assessed with the cytoplasmic H_2O_2-sensitive probe, cyto-roGFP2-Orp1 in adult control heart (E), or hearts with PC-specific (*Dot-Gal4* driver) overexpression of *catalase* (F) or *catalase*^{RNAi} (G). The dotted lines outline the myocardial tubes.

Figure S2

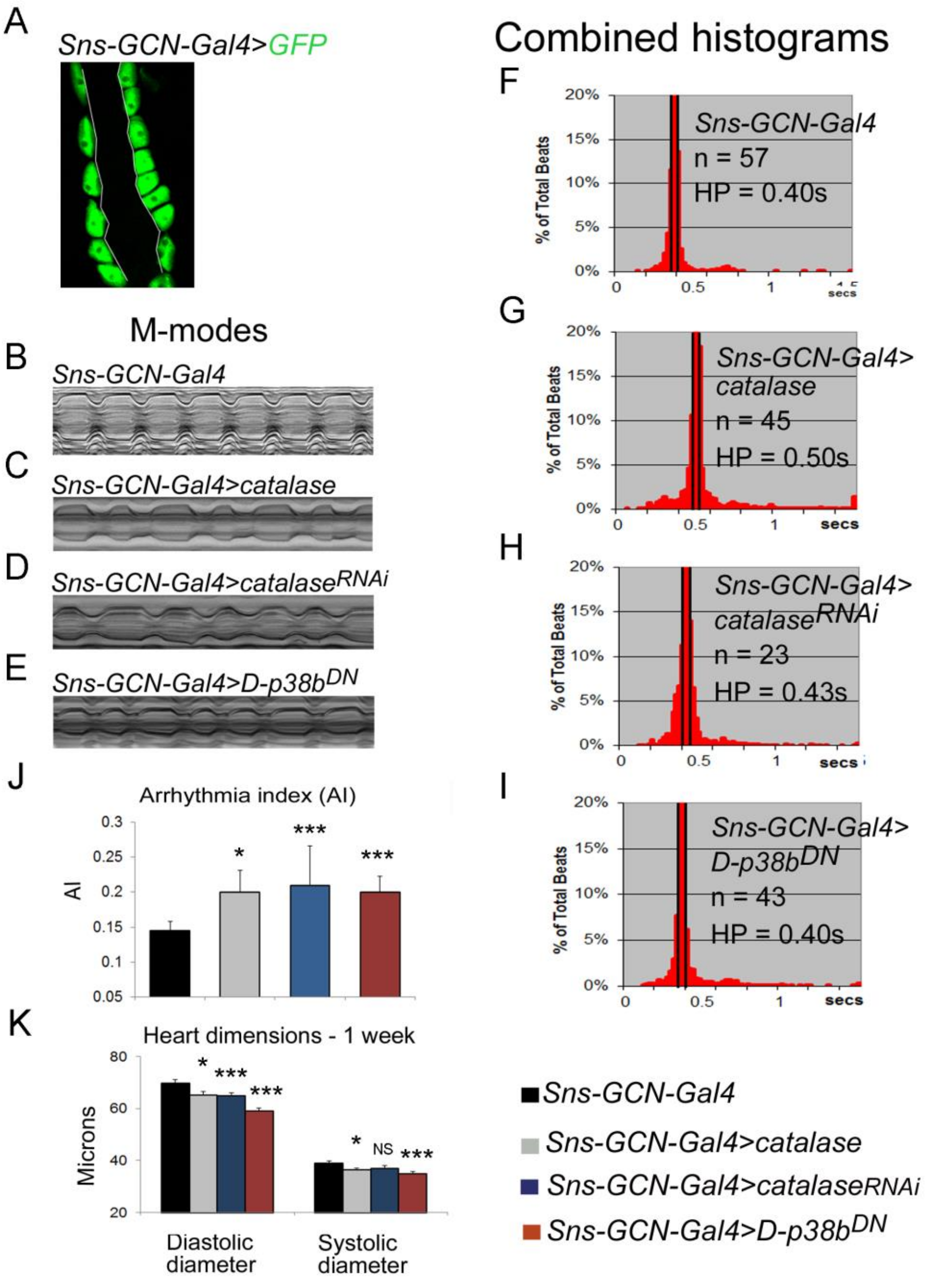

A
Sns-GCN-Gal4>GFP
Combined histograms
F
Sns-GCN-Gal4
n = 57
HP = 0.40s
G
Sns-GCN-Gal4>
catalase
n = 45
HP = 0.50s
H
Sns-GCN-Gal4>
catalaseRNAi
n = 23
HP = 0.43s
I
Sns-GCN-Gal4>
D-p38bDN
n = 43
HP = 0.40s
% of Total Beats
secs
M-modes
B
Sns-GCN-Gal4
C
Sns-GCN-Gal4>catalase
D
Sns-GCN-Gal4>catalaseRNAi
E
Sns-GCN-Gal4>D-p38bDN
J
Arrhythmia index (AI)
AI
K
Heart dimensions - 1 week
Microns
Diastolic diameter
Systolic diameter
NS
Sns-GCN-Gal4
Sns-GCN-Gal4>catalase
Sns-GCN-Gal4>catalaseRNAi
Sns-GCN-Gal4>D-p38bDN

Supplemental Figure 2. Modulation of the ROS–D-p38 signaling pathway in PCs affects cardiac function and morphology, Related to Figure 1.

(A) Confocal image of a fixed heart preparation showing PC-specific (*Sns-GCN-Gal4* driver) *GFP* expression.

(B-E) Representative 5-s M-mode traces showing movement of heart tube walls (Y-axis) versus time (X-axis). Traces are from 1-week-old control flies (B), or flies with PC-specific expression of *catalase* (C), $catalase^{RNAi}$ (D), or $D\text{-}p38^{DN}$ (E).

(F-I) Combined histograms showing the distribution of heart periods (HP). n = number of flies.

(J) Arrhythmia index (AI), with data pooled from 1- and 3-week-old flies.

(K) Heart dimensions of 1-week-old flies. All error bars indicate S.E.M. *$P < 0.05$, ***$P < 0.001$ compared with control *Sns-GCN-Gal4* flies by two-tailed paired *t*-test. NS, not significant.

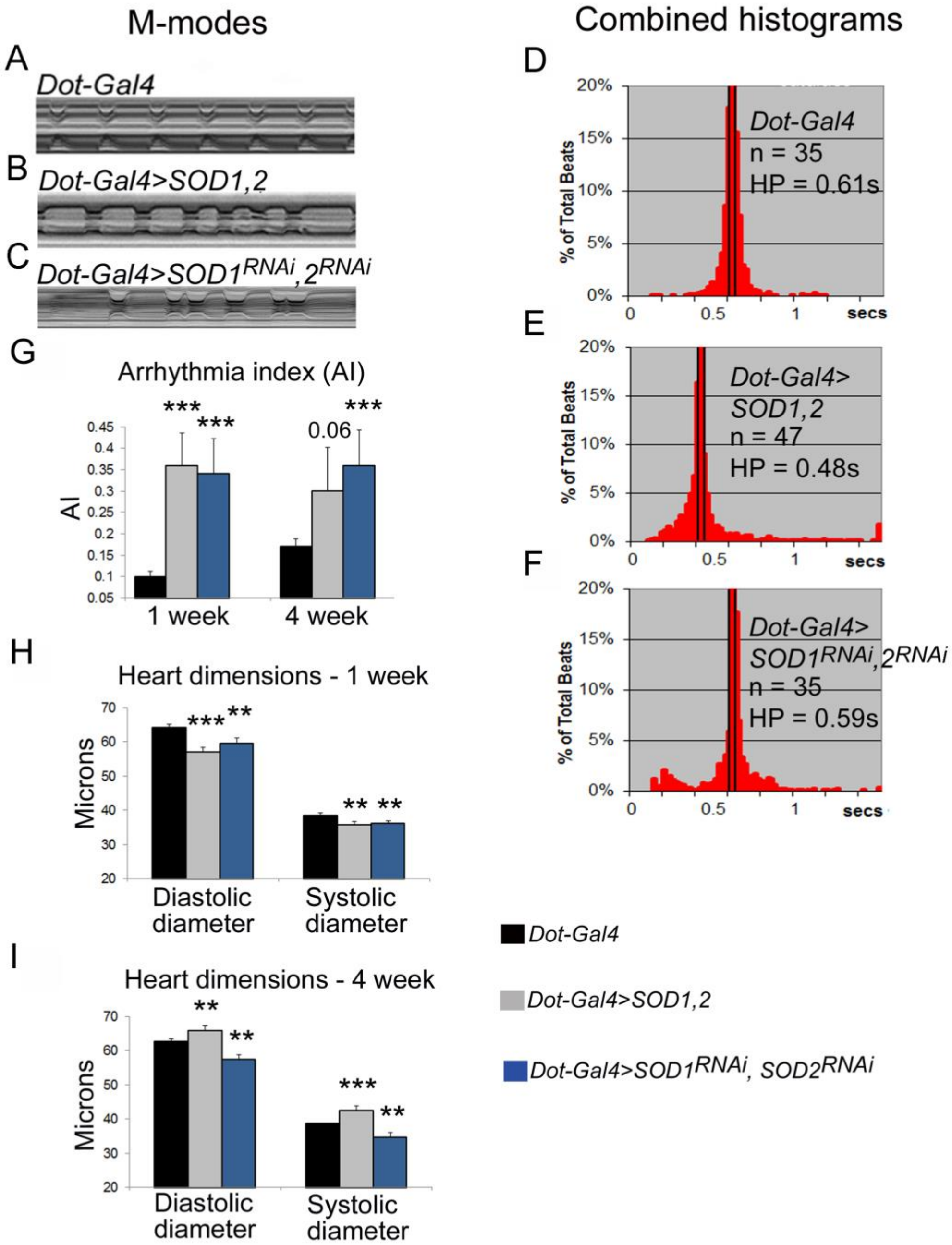
M-modes
A
Dot-Gal4
B
Dot-Gal4>SOD1,2
C
Dot-Gal4>SOD1RNAi,2RNAi
Combined histograms
D
Dot-Gal4
n = 35
HP = 0.61s
E
Dot-Gal4>
SOD1,2
n = 47
HP = 0.48s
F
Dot-Gal4>
SOD1RNAi,2RNAi
n = 35
HP = 0.59s
% of Total Beats
secs
G
Arrhythmia index (AI)
AI
1 week
4 week
0.06
H
Heart dimensions - 1 week
Microns
Diastolic diameter
Systolic diameter
I
Heart dimensions - 4 week
Microns
Diastolic diameter
Systolic diameter
Dot-Gal4
Dot-Gal4>SOD1,2
Dot-Gal4>SOD1RNAi, SOD2RNAi

Figure S3. Modulation of ROS levels in PCs affects cardiac function and morphology, Related to Figure 1.

(A-C) Representative 5-s M-mode traces from 1-week-old control flies (A), or flies with PC-specific co-expression of *SOD1* and *SOD2* (*SOD1,2*) (B) or $SOD1^{RNAi}$ and $SOD2^{RNAi}$ ($SOD1^{RNAi},2^{RNAi}$) (C).

(D-F) Combined histograms showing the distribution of HP. n = number of flies.

(G-I) AI (G) and heart dimensions (H,I) in 1- and 4-week-old flies of the indicated genotypes. All error bars indicate S.E.M. **$P < 0.01$, ***$P < 0.001$ compared with control *Dot-Gal4* flies by two-tailed paired *t*-test.

Figure S4

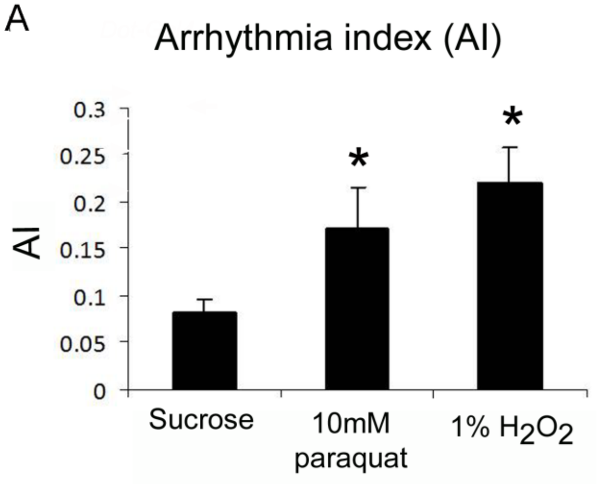
A
Arrhythmia index (AI)
AI
0.3
0.25
0.2
0.15
0.1
0.05
0
*
*
Sucrose
10mM paraquat
1% H2O2

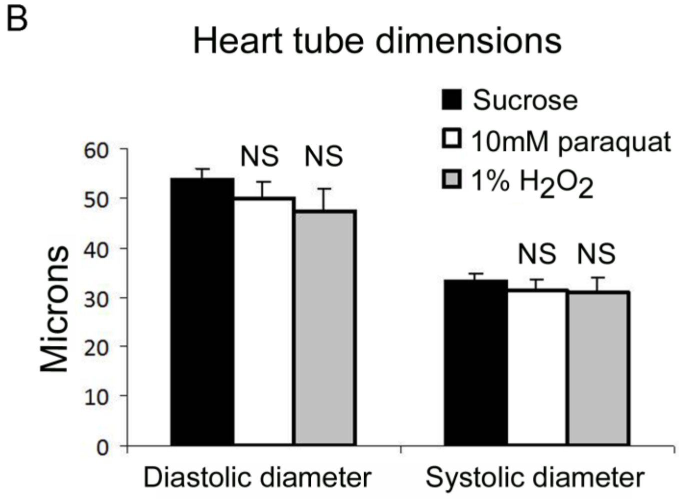
B
Heart tube dimensions
Sucrose
10mM paraquat
1% H2O2
Microns
60
50
40
30
20
10
0
NS NS
NS NS
Diastolic diameter
Systolic diameter

Figure S4. Abnormal cardiac responses after feeding with oxidative stress inducers, Related to Figure 1.

Solutions containing 0.5 M sucrose, sucrose plus paraquat (10 mM), or sucrose plus hydrogen peroxide (1%) were applied to filter pads and placed at the bottom of empty food vials. Newly-eclosed female flies (15–20) were added to the vial and reared for 1 week before heart function analysis. Filter pads were exchanged daily.

(A-B) AI (A) and diastolic and systolic diameters (B) in 1-week-old flies treated with the indicated reagents. All error bars indicate S.E.M. $*P < 0.05$ compared with the sucrose control by two-tailed paired *t*-test. NS, not significant.

Figure S5

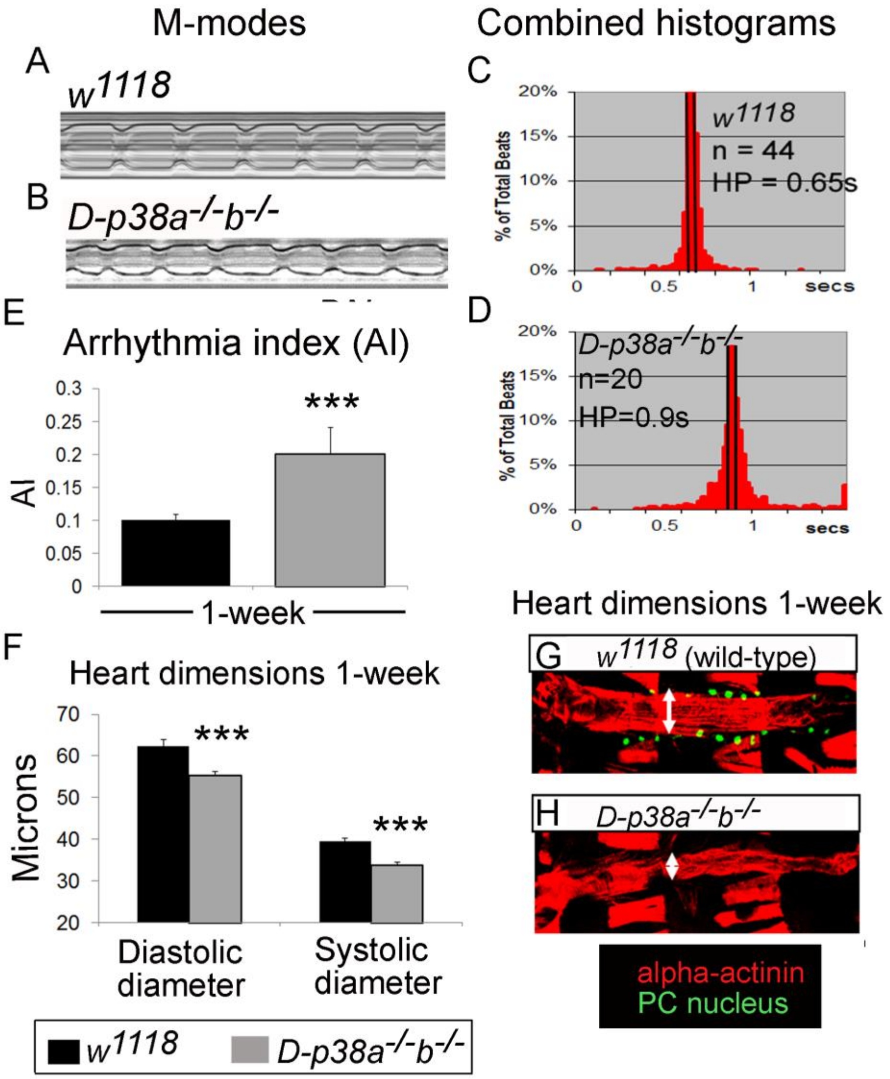

M-modes
Combined histograms
A
w1118
B
D-p38a-/-b-/-
C
20%
15%
10%
5%
0%
% of Total Beats
w1118
n = 44
HP = 0.65s
0
0.5
1
secs
D
D-p38a-/-b-/-
n=20
HP=0.9s
E
Arrhythmia index (AI)
0.3
0.25
0.2
0.15
0.1
0.05
0
AI
***
1-week
F
Heart dimensions 1-week
70
60
50
40
30
20
Microns
***
Diastolic diameter
Systolic diameter
w1118
D-p38a-/-b-/-
Heart dimensions 1-week
G w1118 (wild-type)
H D-p38a-/-b-/-
alpha-actinin
PC nucleus

Figure S5. Loss of *D-p38a* and *D-p38b* elicits cardiac dysfunction in young adult flies, Related to Figure 3.

(A,B) Representative 5-s M-mode traces of wild-type (w^{1118}) and *D-p38a* and *D-p38b* double homozygous mutant ($D\text{-}p38a^{-/-}b^{-/-}$) hearts at 1 week of age.

(C,D) Combined histograms showing the distribution of HP. n = number of flies.

(E,F) AI (E) and average heart dimensions (F). All error bars indicate S.E.M.
***$P < 0.001$ compared with controls by two-tailed paired *t*-test.

(G,H) Representative confocal images of fixed heart preparations stained for alpha-actinin (red) and phosphorylated histone H3 (green), which marks the nuclei of PC only. Images are from wild-type hearts (G) and $D\text{-}p38a^{-/-}b^{-/-}$ hearts (H) at 1 week of age.

Figure S6

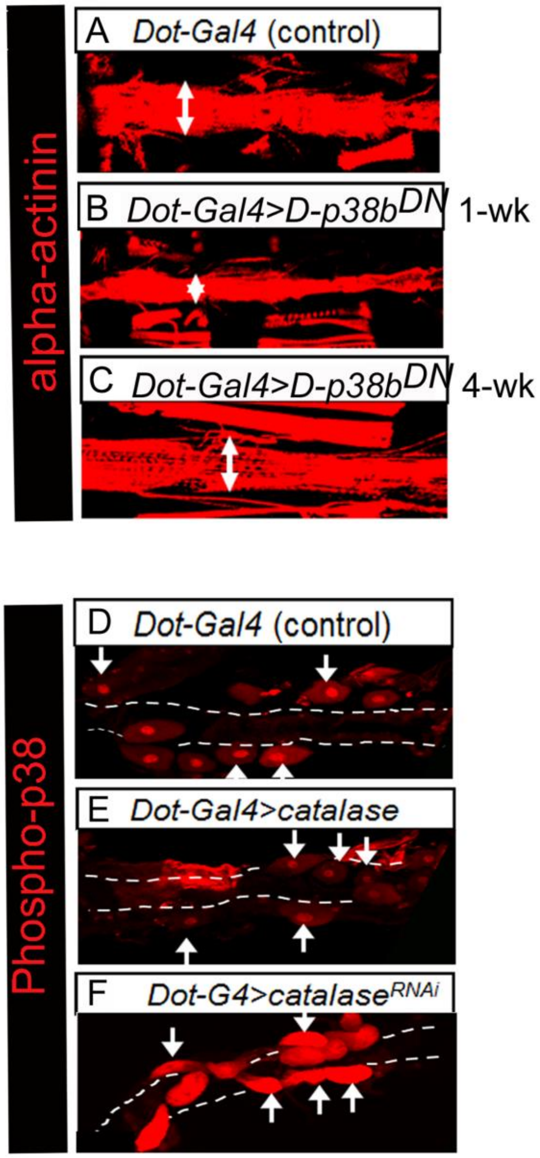
alpha-actinin
A Dot-Gal4 (control)
B Dot-Gal4>D-p38bDN 1-wk
C Dot-Gal4>D-p38bDN 4-wk
Phospho-p38
D Dot-Gal4 (control)
E Dot-Gal4>catalase
F Dot-G4>catalaseRNAi

Figure S6. Inhibition of D-p38b activity in PCs affects heart tube dimensions, and manipulation of the physiological levels of ROS alter the phosphorylation of D-p38 in PCs, Related to Figure 3.

(A-C) Representative confocal images of alpha-actinin staining (red) in fixed heart preparations from control flies (A) or flies with PC-specific overexpression of $D\text{-}p38b^{DN}$ at 1 week of age (B) or 4 weeks of age (C).

(D-F) Representative confocal images of phospho-p38 staining (red) in fixed heart preparations from 1-week-old control flies (D) or flies with PC-specific overexpression of *catalase* (E) or $catalase^{RNAi}$ (F). Anterior is to the left on all images. The dotted lines outline the myocardial tubes.

Figure S7

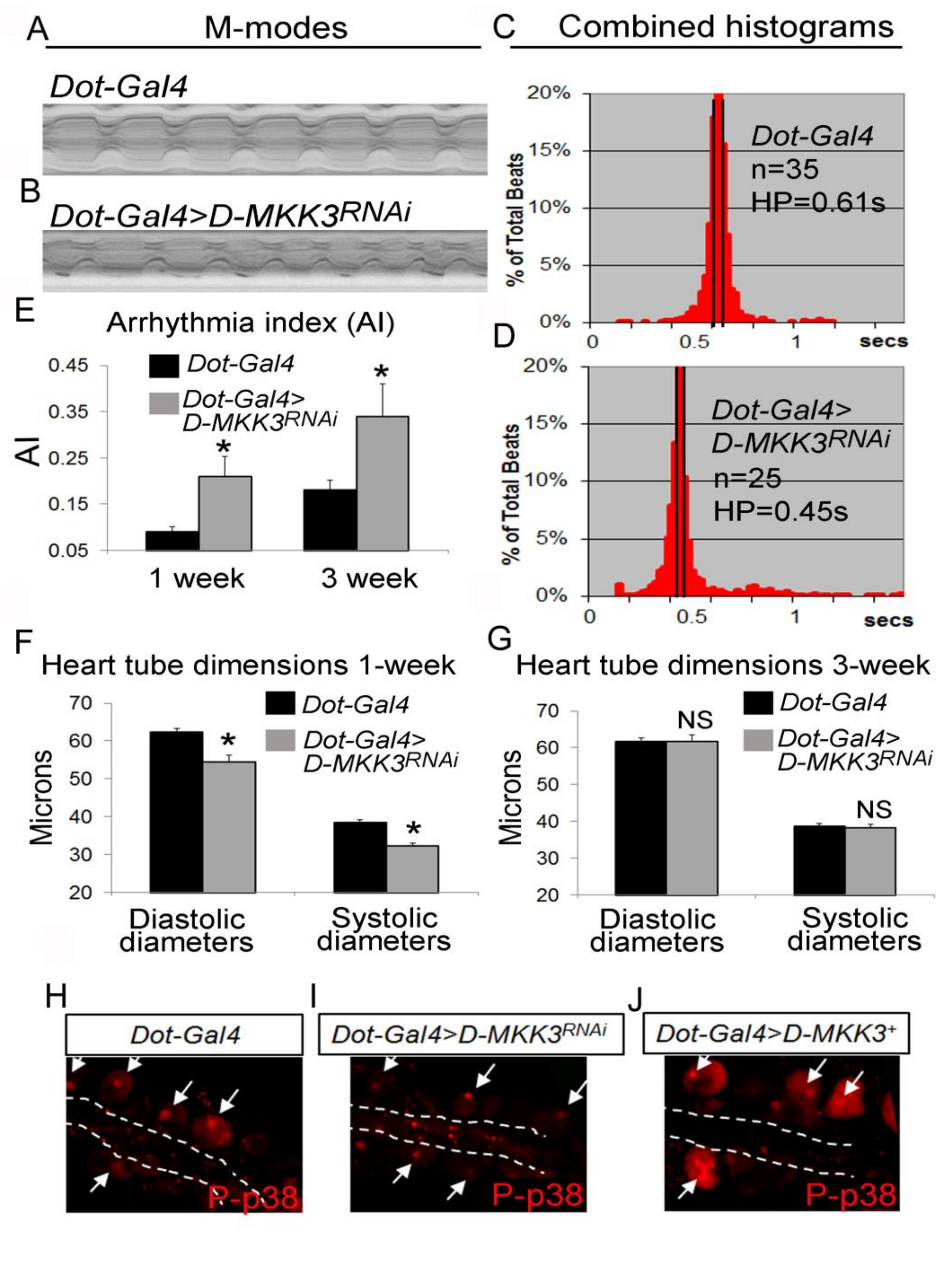

A M-modes
C Combined histograms
Dot-Gal4
B
Dot-Gal4>D-MKK3RNAi
20%
15%
10%
5%
0%
% of Total Beats
Dot-Gal4
n=35
HP=0.61s
0 0.5 1 secs
E Arrhythmia index (AI)
Dot-Gal4
Dot-Gal4> D-MKK3RNAi
AI
0.45
0.35
0.25
0.15
0.05
1 week 3 week
D
Dot-Gal4> D-MKK3RNAi
n=25
HP=0.45s
F Heart tube dimensions 1-week
Microns
70 60 50 40 30 20
Diastolic diameters
Systolic diameters
G Heart tube dimensions 3-week
NS
H Dot-Gal4
I Dot-Gal4>D-MKK3RNAi
J Dot-Gal4>D-MKK3+
P-p38

Figure S7. RNAi-mediated knockdown of *D-MKK3* expression in PCs elicits cardiac dysfunction, Related to Figure 3.

(A,B) Representative 5-s M-mode traces from 1-week-old control flies (A) or flies with PC-specific expression of *DMKK3*^{RNAi} (B).

(C,D) Combined histograms showing the distribution of HP.

(E-G) AI (E) and heart dimensions (F,G) in 1- and 3-week-old flies.). All error bars indicate S.E.M. **P* < 0.05 compared with *Dot-Gal4* control by two-tailed paired *t*-test. NS, not significant.

(H-J) Representative confocal images of phospho-p38 staining (red) in fixed heart preparations from control (H), flies with PC-specific knockdown of *DMKK3* expression via *RNAi*-interference (*DMKK3*^{RNAi}) (I), or flies with PC-specific up-regulation of *DMKK3* expression via overexpressing wild-type *DMKK3* construct (*DMKK3*$^{+}$) (J). The dotted lines outline the myocardial tubes and the arrows indicate PCs. Anterior is to the left.
